# Time-of-Day Effects on Short-Duration Maximal Exercise Performance

**DOI:** 10.1038/s41598-020-66342-w

**Published:** 2020-06-11

**Authors:** Gerardo Gabriel Mirizio, Rodolfo Soares Mendes Nunes, Douglas Araujo Vargas, Carl Foster, Elaine Vieira

**Affiliations:** 1 Muscle Cell Physiology Laboratory, Center of Molecular Studies of the Cell, Institute of Biomedical Sciences, Faculty of Medicine, Universidad de Chile, Santiago, Chile; 2 Postgraduate Program on Physical Education, Universidade Católica de Brasília, Brasília, DF Brazil; 3 Graduate Program on Physical Education, Universidade Católica de Brasília, Brasília, DF Brazil; 4 University of Wisconsin - La Crosse, Department of Exercise and Sport Science, La Crosse, USA

**Keywords:** Physiology, Health care

## Abstract

Time-of-day dependent fluctuations in exercise performance have been documented across different sports and seem to affect both endurance and resistance modes of exercise. Most of the studies published to date have shown that the performance in short-duration maximal exercises (i.e. less than 1 min - e.g. sprints, jumps, isometric contractions) exhibits diurnal fluctuations, peaking between 16:00 and 20:00 h. However, the time-of-day effects on short duration exercise performance may be minimized by the following factors: (1) short exposures to moderately warm and humid environments; (2) active warm-up protocols; (3) intermittent fasting conditions; (4) warming-up while listening to music; or (5) prolonged periods of training at a specific time of day. This suggests that short-duration maximal exercise performance throughout the day is controlled not only by body temperature, hormone levels, motivation and mood state but also by a versatile circadian system within skeletal muscle. The time of day at which short-duration maximal exercise is conducted represents an important variable for training prescription. However, the literature available to date lacks a specific review on this subject. Therefore, the present review aims to (1) elucidate time-of-day specific effects on short-duration maximal exercise performance and (2) discuss strategies to promote better performance in short-duration maximal exercises at different times of the day.

## Introduction

Circadian rhythms are responsible for temporal regulation of numerous physiological phenomena in the human body. At a molecular level, circadian rhythms are defined as a function of clock gene expression levels over a 24-hour period^1^. Clock gene expression levels throughout the day set up the pace of sleep-wake cycles, hunger, hormone production, body temperature, as well as other physiological functions^2^. In mammals, biological rhythms are driven by a central pacemaker located in the suprachiasmatic nuclei (SCN) of the hypothalamus. It consists of approximately 20 000 neurons that exhibit independent rhythms of firing rate and gene expression^3^. The core clock system of the SCN works as a self-sustained transcriptional/translational feedback loop, involving a set of four integral proteins that act as activators or repressors within the system^4^. In addition, there is a group of kinases and phosphatases that regulate their localization and stability^5^. Apart from the core clock system in the SCN, circadian clocks and clock-controlled output genes (CCGs) are also present in peripheral tissues such as the liver, heart, kidney, pancreas, adipose tissue and skeletal muscle^6–12^. In this sense, the main synchronizer of the internal clock is solar light^13^, but it is known now that other non-photic stimuli such as feeding, social contact or physical exercise do also modulate the transcriptional activity of clock genes^14–17^. Likewise, internal clocks also play important roles on human behavior, accounting for variations in resting levels of neuromuscular, sensory-motor and cognitive performance throughout the day^18–21^. Recently, high-throughput transcriptomic and metabolomic analyses in mice have demonstrated that the time of day is a crucial factor to amplify the effect of exercise on systemic energy homeostasis and metabolic pathways within skeletal muscle^22^. In addition, exercise capacity exhibit diurnal fluctuations in mice and humans between the early and late part of their active phase^23^, which suggests that the time of day is a major modifier of exercise capacity.

Time-of-day dependent fluctuations in exercise performance have been documented across different sports and seem to affect both endurance and resistance modes of exercise^24–30^. Whereas differences in endurance exercise performance might be as large as 26%^25^, strength performance may vary as much as 41% throughout the day^30^. Short-duration maximal exercises (i.e. less than 1 min - e.g. all-out sprints, maximal jumps or isometric contractions) comprise both modes of exercise and are characterized by higher locomotor requirements^31^ and anaerobic energy contributions^32^ than longer exercises. Thus, they represent a robust model to explore time-of-day effects on the musculoskeletal system. Previous studies have shown that performance in short-duration maximal exercises exhibits time-of-day dependent fluctuations with amplitudes up to 29.4% between the morning and evening hours^24,33–45^. Considering the large variation in short-duration maximal exercise performance throughout the day, it is clear that the time of day at which our athletes train or compete is not trivial. However, the literature available to date lacks a specific review on this subject. Therefore, the present review aims to (1) elucidate time-of-day specific effects on short-duration maximal exercise performance and (2) discuss strategies to promote better performance in short-duration maximal exercises at different times of the day.

## Methods

The present article provides an up-to-date review of the literature about the effects of time of day on short-duration maximal exercise performance. Articles were searched via three online databases (PubMed, PubMed Central and Google Scholar; 1924–2019). The literature search strategy included a combination of free terms using the Boolean operators “AND” and “OR”. The free terms used in the search were: time-of-day, circadian, chronotype, strength, neuromuscular, resistance, endurance, aerobic, anaerobic, short-duration, performance, isometric, isokinetic, dynamic, morning, evening, afternoon and night. The full search term strategy that was used for each scientific database is outlined in the supplementary material.

**Figure 1:**
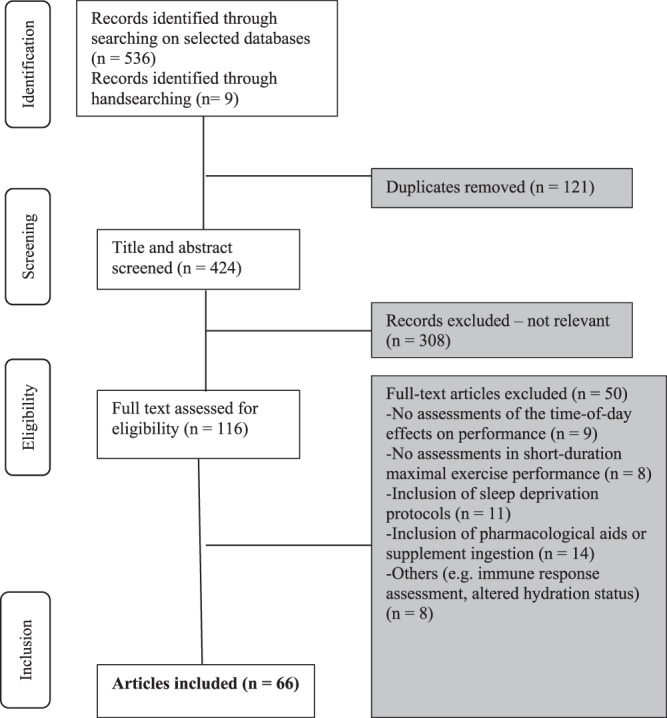
Flow diagram of the article selection process.

The studies retrieved from the databases that fit the inclusion criteria were imported to EndNote Web Software (Thomson Reuters, New York, USA) where duplicated articles were identified and excluded. Then, the titles and abstracts of the remaining articles were reviewed. Articles were excluded at this initial screening phase if they did not mention any specific effect of time of day on exercise performance. If this information could not be ascertained from the title or abstract, the article’s full text was reviewed in the next screening phase to determine whether it fitted the eligibility criteria. After the initial title/abstract screening process, the full texts of all the remaining articles were assessed to select those that fitted the inclusion criteria. The inclusion criteria that were applied to original articles included: (1) the study explored the effects of time of day (over a 24-hour period) on one or more aspects of exercise performance; (2) the study assessed time-of-day effects on isometric, isokinetic and dynamic short-duration maximal (i.e. less than 1 min) exercises (3) the study assessed the effects of either acute or chronic interventions on time-of-day dependent fluctuations in exercise performance; (4) the study assessed either direct (i.e. peak and mean power, isokinetic peak torque, total work, jump height) or indirect (i.e. RPE, neuromuscular efficiency, markers or muscle injury) features of exercise performance; (5) the study was published in indexed journals and was available in English. As a result, a total of 66 original articles were selected out of 545 initial results. A flow diagram of the article selection process is also outlined in the Fig. 1.

## Results

A total of 66 articles were selected and then divided into dynamic and/or isometric/isokinetic short-duration maximal exercises. Forty-four articles assessed time-of-day effects on dynamic short-duration maximal exercise performance, including swimming, tennis, jumping, cycling, sprinting and resistance exercises (Table 1). On the other hand, 32 articles assessed time-of-day effects on isometric and isokinetic exercise performance, including electrically induced contractions, reflex contractions, maximal and submaximal voluntary isometric contractions, isokinetic leg contractions and magnetic stimulation of the cortex (Table 2). Most articles compared time-of-day effects on short-duration maximal exercise performance under acute exercise conditions; however, 11 articles assessed time-of-day effects on short-duration maximal exercise performance after a period of training (from 5 to 10 weeks) (Tables 1 and 2).

**Table 1:** Summary of the literature about time-of-day effects on dynamic short-duration maximal exercise performance.

Author(s)	Date	Protocol	Training period	Number of participants	Characteristics of the participants	Main Results (% change of peak performance time vs. other assessment time-points)	Time-of-day effect?	Peak performance time	Assessment Time-points
Aloui *et al*.^85^	2013	Repeated Sprint Ability^1^ test	—	n = 12 males	Recreationally trained soccer players	↑ 5.4% in P_peak_ during the first sprint ↔ time-of-day dependent variations in P_peak_ after 2 and 4 weeks of intermittent-fasting^3^ conditions	Yes	17:00–19:00 h	07:00–09:00 and 17:00–19:00 h
Arnett^47^	2002	All-out swim trials	—	n = 6 males n = 4 females	Competitive young swimmers	↑ 0.9% in all-out (100-yd) swimming performance ↔ time-of-day variation in body temperature, but not evening superiority in all-out (100-yd) swimming performance when high-volume^4^ warm up conditions were imposed	Yes	17:00 h	06:15 and 17:00 h
Atkinson and Speirs^37^	1998	Tennis services	—	n = 3 males n = 3 females	Competitive young adult tennis players	↑ 3.9% in first serves speed (but lower serve accuracy) ↔ second serves speed (nor serve accuracy) throughout the day	Yes	18:00 h	09:00, 14:00 and 18:00 h
Baxter and Reilly^48^	1983	All-out swim trials	—	n = 4 males n = 10 females	Competitive young swimmers	↑ 3.5% in 100 m swimming performance	Yes	17:00 h	06:30, 09:00, 13:30, 17:00 and 22:00 h
Belkhir *et al*.^86^	2019	5-m shuttle run test	—	n = 12 males	Competitive soccer players	↑ 3.6% in total distance and 13.1% in higher distance during the 5-m shuttle run test under warm-up without music conditions ↑ 3.5–6.9% in anaerobic performance at 07:00 h under neutral and self-selected music warm-up conditions ↑ 3.6–5.3% in anaerobic performance at 17:00 h under neutral and self-selected music warm-up conditions	Yes	17:00 h	07:00 and 17:00 h
Bernard *et al*.^38^	1998	Force-Velocity and Multi-Jump tests	—	n = 23 males	Physical Education students	↑ 3.5% in maximal anaerobic power for cycling throughout the day ↑ 5–6.6% in maximal anaerobic power for jumping throughout the day	Yes	14:00 and 18:00 h	09:00, 14:00 and 18:00 h
Blonc *et al*.^46^	2010	Squat Jump, Countermovement Vertical Jump and Cycle sprints^2^ tests	—	n = 12 males n = 4 females	Physical Education students	↔ SJ, CMJ and Cycle sprints performances throughout the day	No	—	07:00–09:00 and 17:00–19:00 h
Castaingts *et al*.^55^	2004	Drop Jump test	—	n = 11 males	N/A	↑ 10.9% in jump height and 15.7% in NME (mechanical performances/EMG recordings) ↔ reflex excitability throughout the day	Yes	18:00 h	06:00 and 18:00 h
Chtourou *et al*.^13^	2012	Wingate anaerobic test	—	n = 12 males	Physical Education students	↑ 5.3% in P_peak_ and 3.3% in P_mean_ ↓ 48.9% time-of-day dependent variations in P_peak_ after a music-coupled warm-up^5^ ↔ time-of-day dependent variations in P_mean_ after a music-coupled warm-up^5^	Yes	17:00 h	07:00 and 17:00 h
Chtourou *et al*.^30^	2012	Wingate anaerobic test, Squat Jump and Countermovement Vertical Jump tests	12 weeks of training + 2 weeks of tapering at a specific time of day	n = 31 males	Physical Education students	↑ 3.3% in P_peak_ and 3% in P_mean_ before the training period ↑ 7.2% in SJ and 5.9% in CMJ heights before the training period ↔ SJ, CMJ and Wingate test performance throughout the day after 12 weeks of training at a specific time of day ↑ 4–7.3% in anaerobic performance at 07:00 h after 12 weeks of training at a specific time of day ↑ 4–5.3% in anaerobic performance at 17:00 h after 12 weeks of training at a specific time of day	Yes	17:00 h	07:00 and 17:00 h
Chtourou *et al*.^50^	2012	Wingate anaerobic test	—	n = 10 males	Competitive young football players	↑ 3.14% in P_peak_ and 2.9% in P_mean_ ↔ time-of-day dependent variations in P_peak_ and P_mean_ after 2 and 4 weeks of intermittent-fasting^3^ conditions	Yes	17:00 h	07:00 and 17:00 h
Chtourou *et al*.^24^	2011	Wingate anaerobic test	—	n = 22 males	Physical Education students	↑ 2.6% in P_peak_ and 2.3% in P_mean_ ↑ 4% in NME during the first (firsts 20 s) but not second (lasts 10 s) of the Wingate anaerobic test.	Yes	17:00 h	07:00 and 17:00 h
Giacomoni, Billaut and Falgairette^33^	2006	Repeated Sprint Ability^6^ test	—	n = 12 males	Physically active and healthy adults	↔ biomechanical indices of neuromuscular performance (P_peak,_ total mechanical work, peak pedaling rate) throughout the day	No	—	08:00–10:00 and 17:00–19:00 h
Hammouda *et al*.^58^	2011	Repeated Sprint Ability^1^ test	—	n = 12 males	Well-trained young football players	↑ 5.4% in sprint 1 and 4.4% in sprint 2 P_peak_ ↑ 1.6% in P_peak_ sprint decrease ↑ 0.7% in RPE ↑ 12.3% in resting homocysteine levels and 17.6–35.4% in biological markers of muscle injury at 17:00–18:30 h ↓ 4.4–12.9% in biomarkers of antioxidant status’ resting levels at 17:00–18:30 h	Yes	17:00–18:30 h	07:00–08:30 h and 17:00–18:30 h
Hammouda *et al*.^27^	2012	Wingate anaerobic test	—	n = 15 males	Well-trained young football players	↑ 3.6% in P_peak_ and 2.8% in P_mean_	Yes	17:00–18:30 h	07:00–08:30 h and 17:00–18:30 h
Hill *et al*.^51^	1992	All-out cycle ergometer test	—	n = 8 males n = 6 females	N/A	↑ 9.6% in total work in the afternoon compared to the morning ↑ 5.1% in anaerobic power in the afternoon compared to the morning	Yes	N/A	N/A
Javierre *et al*.^67^	1996	80 m all-out sprint performance	—	n = 8 males	Competitive sprinters	↑ sprint performance (faster sprint times) at 19:00 h ↑ 2.7–4.1% in sprint performance at 17:00 h when sleep/wake cycles and mealtimes were advanced for two hours (vs sprint performance at 17:00 h on a control day) ↑ 1.7–2.3% in sprint performance at 21:00 h when sleep/wake cycles and mealtimes were delayed for two hours (vs sprint performance at 21:00 h on a control day)	Yes	19:00 h	09:00, 11:00, 13:00, 15:00, 17:00, 19:00, 21:00 and 23:00 h
Krčmárová *et al*.^63^	2018	Dynamic leg press and seated-row 6-repetition maximum (6RM) and functional capacity^7^ tests	12 weeks of training at a specific time of day	n = 31 females	Untrained healthy older adults	↔ strength performance with time-of-day after 12 weeks of training at a specific time of day	No	—	07:30 and 18:00 h
Küüsmaa *et al*.^64^	2016	Dynamic leg press test of 1-repetition maximum (1RM)	24 weeks of training at a specific time of day	n = 42 males	Untrained healthy youngsters	↔ 1RM gains after 24 weeks of training at a specific time of day	No	—	06:30–09:30 and 16:30–19:30 h
Lericollais *et al*.^28^	2009	Wingate anaerobic test	—	n = 16 males	Competitive cyclists	↑ 8.2% in P_peak_ and 7.8% in P_mean_	Yes	18:00 h	06:00 and 18:00 h
Lericollais *et al*.^52^	2011	Wingate anaerobic test	—	n = 20 males	Physically active and healthy adults	↑ 6.6% in P_peak_, 3.3% in P_mean30s_ and 2.7% in P_mean60s_	Yes	18:00 h	06:00 and 18:00 h
López-Samanes *et al*.^40^	2017	Serve velocity/accuracy, Countermovement Vertical Jump, Agility T-test^8^ and 10-m sprint tests	—	n = 13 males	Competitive tennis players	↑ 4% in serve velocity/accuracy test ↑ 4.5% in CMJ ↑ 2.1% in Agility T-test ↑ 2.7% in 10-m RUN performances	Yes	16:30 h	09:00 and 16:30 h
Melhim^53^	1993	Wingate anaerobic test	—	n = 13 females	Physical Education students	↑ 7% in P_peak_ and 15–16% in P_mean_	Yes	15:00 and 21:00 h	03:00, 09:00, 15:00 and 21:00 h
Pallarés *et al*.^49^	2014	Bench Press, Countermovement Vertical Jump, Crank-arm (10 s Wingate test), and 25 m swimming freestyle tests	—	n = 6 males n = 6 females	Well-trained junior swimmers	↑ 5.1% in bench press maximum strength and muscle power ↑ 1.7% in 25-m swimming performance ↑ 5.8% in CMJ height	Yes	18:00 h	10:00 and 18:00 h
Pullinger *et al*.^87^	2019	Handgrip strength, Bench Press and Back Squat tests	—	n = 10 males	Resistance trained young adults	↑ 4.6% in right and. 6.5% in left handgrip strength ↑ 3.3% in bench press and 2.6% in back squat average force ↑ 13.6% in bench press and 11.5% in back squat peak velocity ↓ 17% in bench press and 14.1% in back squat time to peak velocity	Yes	17:30 h	07:00 and 17:30 h
Pullinger *et al*.^89^	2018	All-out sprint^9^ test	—	n = 12 males	Resistance trained young adults	↑ 7.8–8.3% in total sprint distance, average mean and average velocity	Yes	17:30 h	07:00 and 17:30 h
Racinais, Blonc and Hue^59^	2005	All-out sprint^2^ test	—	n = 8 males	Physical Education students	↑ 4.5% in P_max_ and 3.8% in F_max_ ↑ 3.7% in P_max_ and 3.1% in V_max_ after active warm-up^11^ vs. passive^12^ warm-up conditions at any time of day	Yes	17:00–19:00 h	07:00–09:00 and 17:00–19:00 h
Racinais, Hue and Blonc^56^	2004	Squat-Jump, Countermovement Vertical Jump and all-out sprint^2^ tests	—	n = 12 males	Physical Education students	↑ 3.1–3.7% in CMJ, SJ and cycling sprint performances ↔ muscular performance throughout the day when moderately warm and humid^13^ conditions were imposed	Yes	17:00–19:00 h	07:00–09:00 and 17:00–19:00 h
Racinais *et al*.^62^	2004	Countermovement Vertical Jump and Force-Velocity tests	—	n = 15 males n = 8 females	Physical Education students	↔ maximal anaerobic power throughout the day when warm and humid^14^ conditions were imposed	No	—	08:00, 13:00 and 17:00 h
Racinais *et al*.^61^	2005	Repeated Sprint Ability^1^ test	—	n = 9 males	Physical Education students	↑ 5.3% in P_peak_ during the first sprint ↑ 12% in blood lactate concentration at the end of the RSA test	Yes	17:00–19:00 h	07:00–09:00 and 17:00–19:00 h
Racinais *et al*.^2^	2006	Countermovement Vertical Jumps, all-out^2^, and Isokinetic leg strength (knee flexors and extensors) tests	—	n = 9 males	Physically active and healthy adults	↔ CMJ, sprint and isokinetic torque performances throughout the day when moderately warm and humid^15^ conditions were imposed	No	—	07:00–09:00 and 17:00–19:00 h
Racinais *et al*.^60^	2009	All-out sprint^2^ test	—	n = 7 males	Physical Education students	↑ 12% in P_max_, 5% in F_max_, and 6% in V_max_ ↔ P_max_, F_max_, and V_max_ throughout the day when local pre-cooling^16^ or -heating^17^ conditions were imposed	Yes	17:00–19:00 h	07:00–09:00 and 17:00–19:00 h
Robertson *et al*.^88^	2018	Bench Press and Back Squat tests	—	n = 30 males	Resistance trained young adults	↑ 1.9% in bench press and 2.5% in back squat average force ↑ 8.3% in bench press and 12.7% in back squat peak velocity ↓ 16.6% in bench press and 9.8% in back squat time to peak velocity	Yes	17:30 h	07:00 and 17:30 h
Sedliak *et al*.^41^	2008	Squat-Jump test	—	n = 32 males	Physically active and healthy adults^10^	↑ 4.6–5.6% in power output during the concentric phase of loaded SJ	Yes	12:00–13:00, 17:00–18:00 and 20:30–21:30 h	07:00–08:00, 12:00–13:00, 17:00–18:00 and 20:30–21:30 h
Sedliak *et al*.^66^	2008	One-repetition maximum (1RM) half-squat test	10 weeks of training at a specific time of day	n = 34 males	Physically active and healthy adults^10^	↔ Half-Squat 1RM peak torque after 10 weeks of training at a specific time of day	No	—	09:00–16:00 h
Sedliak *et al*.^65^	2009	One-repetition maximum (1RM) half-squat test	10 weeks of training at a specific time of day	n = 24 males	Physically active and healthy^10^	↔ Half-Squat 1RM peak torque after 10 weeks of training at a specific time of day	No	—	09:00–16:00 h
Souissi *et al*.^35^	2010	Squat-Jump, Five-Jump and Wingate anaerobic tests	—	n = 20 males	Untrained healthy boys	↑ 3.5% in SJ and 5% in Five-Jump test performances ↑ 5.5% in P_peak_ and 6% in P_mean_ during the Wingate anaerobic test	Yes	14:00 and 18:00 h	08:00, 14:00 and 18:00 h
Souissi *et al*.^36^	2012	Squat-Jump, Countermovement Vertical Jump and Wingate anaerobic tests	6 weeks of training at a specific time of day	n = 24 males	Untrained healthy boys	↑ 11.5% in SJ and 10.7% in CMJ performances before the training period ↑ 6.3% in P_peak_ and 5% in P_mean_ during the Wingate anaerobic test before the training period ↔ SJ, CMJ, P_peak_ and P_mean_ throughout the day in the morning-training group after 6 weeks of training at a specific time of day	Yes	17:00 h	07:00–08:00 and 17:00–18:00 h
Souissi *et al*.^42^	2010	Wingate anaerobic tests	—	n = 12 males	Physical Education students	↑ 6.8% in P_peak_ and 4.1% in P_mean_ after 5-min active warm up conditions ↑ 3.7% in P_peak_ and 1.6% in P_mean_ after 15-min active warm up conditions ↓ 45.5% time-of-day differences in P_peak_ and 60.9% P_mean_ in the morning after longer (15-min) than shorter (5-min) active warm-ups	Yes	18:00 h	08:00 and 18:00 h
Souissi *et al*.^54^	2002	Wingate anaerobic test	6 weeks of training at a specific time of day	n = 14 males	Physical Education students	↑ 8.4% and 4.1% in P_peak_ in the morning- and evening-training groups before the training period ↔ P_peak_ throughout the day in the morning-training group after 6 weeks of training at a specific time of day	Yes	17:00–18:00 h	07:00–08:00 and 17:00–18:00 h
Souissi *et al*.^43^	2004	Force-Velocity and Wingate anaerobic tests	—	n = 19 males	Physical Education students	↑ 7% in P_max_, 7.6% in P_peak_ and 11.3% in P_mean_	Yes	17:10–18:00 h^*^	02:00, 06:00, 10:00, 14:00, 18:00 and 22:00 h
Taylor *et al*.^57^	2011	Countermovement Vertical Jump tests	—	n = 8 males	Recreationally trained adults^18^	↑ 6.4% in CMJ height (cm) ↔ time-of-day dependent variations in CMJ performance when extended warm-up^19^ conditions were imposed in the morning	Yes	16:00 h	08:00 and 16:00 h
West *et al*.^90^	2014	Countermovement Vertical Jump tests	—	n = 16	Elite rugby union seven players	↑ 3.1% in CMJ P_peak_	Yes	17:00 h	10:00 and 17:00 h
Zarrouk *et al*.^44^	2012	Repeated Sprint Ability^1^ test	—	n = 12 males	Physical Education students	↑ Total work, P_peak_ and %P_peak_ decrement during the first 3 sprints ↔ EMG throughout the day	Yes	18:00 h	06:00 and 18:00 h

RPE rating of perceived exertion, P_max_ Maximal Power, P_peak_ Peak Power, P_mean_ Mean Power, F_max_ Maximal Force, V_max_ Maximal Velocity, CMJ countermovement Vertical jump, SJ Squat Jump, MCV Maximal Voluntary Isometric Contraction, NME Neuromuscular efficiency, EMG Electromyographic activity, N/A Not available, ^1^ i.e. 5 × [6 s of maximal cycling sprint + 24 s of rest], ^2^ i.e. 3 × [7 s of maximal cycling sprint + 5 min of rest], ^3^ i.e. 15–16 h starvation/day; from ≈ 04:00 h till ≈ 19:00 h, ^4^ i.e. 200% of standard warm-up times, ^5^ i.e. 10-min warm up while listening high tempo music (>120 to 140 bpm) through headphones.

RPE rating of perceived exertion, P_max_ Maximal Power, P_peak_ Peak Power, P_mean_ Mean Power, P_mean30s_ Mean Power at 30s, P_mean60s_ Mean Power at 60s, F_max_ Maximal Force, V_max_ Maximal Velocity, CMJ countermovement Vertical jump, 10-m RUN 10-m sprint, SJ Squat Jump, MCV Maximal Voluntary Isometric Contraction, NME Neuromuscular efficiency, EMG Electromyographic activity, N/A Not available, ^1^ i.e. 5 × [6 s of maximal cycling sprint + 24 s of rest], ^6^ i.e. 10 ×[6 s of maximal cycling sprint + 30 s of rest]), ^3^ i.e. 15–16 h starvation/day; from ≈ 04:00 h till ≈ 19:00 h.

RPE rating of perceived exertion, P_max_ Maximal Power, P_peak_ Peak Power, P_mean_ Mean Power, P_mean30s_ Mean Power at 30s, P_mean60s_ Mean Power at 60s, F_max_ Maximal Force, V_max_ Maximal Velocity, CMJ countermovement Vertical jump, 10-m RUN 10-m sprint, SJ Squat Jump, MCV Maximal Voluntary Isometric Contraction, NME Neuromuscular efficiency, EMG Electromyographic activity, ^7^ i.e. 30-second chair stands and arm curl test, Timed Up and Go, ^8^ i.e. all-out running test with lateral and front-to-back movements, ^9^ i.e. 3 ×[3 s of maximal running sprint + 30 s of rest].

RPE rating of perceived exertion, P_max_ Maximal Power, P_peak_ Peak Power, P_mean_ Mean Power, F_max_ Maximal Force, V_max_ Maximal Velocity, CMJ countermovement Vertical jump, SJ Squat Jump, MCV Maximal Voluntary Isometric Contraction, NME Neuromuscular efficiency, EMG Electromyographic activity, ^1^ i.e. 5 ×[6 s of maximal cycling sprint + 24 s of rest], ^2^ i.e.3 × [7 s of maximal cycling sprint + 5 min of rest], ^10^ i.e. no medication within the last14 days, a non-smoker, regular sleep pattern with sleep duration ranging from 6 to 9 h per night and regular physical activity not more than once a week, ^11^ i.e. 12 min of pedaling at 50% of V˙O2max interspersed with three brief accelerations of 5 s, ^12^ i.e. 3 min of pedaling at 70 rpm at 50% of V˙O2max, ^13.^ i.e. 60 min of resting in a room at 29 °C, 70% relative humidity, ^14^ i.e. 60 min of resting in a room at 28.1 °C, 62.6% relative humidity, ^15^ i.e. 60 min of resting in a room at 24 °C, 70% relative humidity, ^16^ i.e. 30 min exposure to a cold bath at 16 °C, ^17^ i.e. 30 min exposure to a bath at 38 °C.

RPE rating of perceived exertion, P_max_ Maximal Power, P_peak_ Peak Power, P_mean_ Mean Power, F_max_ Maximal Force, V_max_ Maximal Velocity, CMJ countermovement Vertical jump, SJ Squat Jump, MCV Maximal Voluntary Isometric Contraction, NME Neuromuscular efficiency, EMG Electromyographic activity, ^1^ i.e. 5 ×[6 s of maximal cycling sprint + 24 s of rest], ^10^ i.e. no medication within the last14 days, a non-smoker, regular sleep pattern with sleep duration ranging from 6 to 9 h per night and regular physical activity not more than once a week,^18^ i.e. a minimum of 6 months resistance training history ^19^ i.e. 20 min general warm-up on a stationary bike at 150–200 W prior to completion of the control warm-up, resulting in a whole-body temperature increase of 0.3 ± 0.2 °C, ^*^estimated acrophase by cosinor analysis.

**Table 2:** Summary of the literature about time-of-day effects on isometric and isokinetic exercise performance.

Author(s)	Date	Protocol	Training period	Number of participants	Characteristics of the participants	Main Results (% change of peak performance time vs. other assessment time-points)	Time-of-day effect?	Peak performance time	Assessment Time-points
Aloui *et al*.^85^	2013	Maximal voluntary isometric knee extensions	—	n = 12 males	Recreationally trained soccer players	↑ 8.9% in MVC peak torque ↔ time-of-day dependent variations in MVC peak torque after 2 and 4 weeks of intermittent-fasting^2^ conditions	Yes	17:00–19:00 h	07:00–09:00 and 17:00–19:00 h
Callard *et al*.^80^	2000	Maximal voluntary isometric knee extensions	—	n = 6 males	Competitive cyclists	↑ 6% in MVC peak torque under resting conditions ↑ 7.8% in MVC peak torque under 24-h cycling^3^ conditions ↑ EMG activity at 13:00, 17:00 and 21:00 h	Yes	19:10–19:30 h^*^	00:01, 05:00, 09:00, 13:00, 17:00 and 21:00 h
Castaingts *et al*.^55^	2004	Electrically induced, reflex and maximal and submaximal voluntary isometric contractions	—	n = 11 males	N/A	↑ 17.6% in NME (in electrically induced contraction conditions) ↔ NME (in maximal voluntary isometric contraction conditions) throughout the day ↔ Reflex excitability throughout the day	Yes	18:00 h	06:00 and 18:00 h
Chtourou *et al*.^30^	2012	Maximal voluntary isometric knee extensions	12 weeks of training + 2 weeks of tapering at a specific time of day	n = 31males	Physical Education students	↑ 10.8% in MVC peak torque before the training period ↔ MVC peak torque throughout the day in the morning-training group after 12 weeks of training + 2 weeks of tapering at a specific time of day ↔ magnitude of MVC strength gains after 12 weeks of training + 2 weeks of tapering at a specific time of day	Yes	17:00 h	07:00 and 17:00 h
Edwards *et al*.^76^	2013	Handgrip strength, isokinetic leg strength^1^, and maximal voluntary isometric contractions	—	n = 10 males	Physically active and healthy adults	↑ 3.3% in grip strength performance ↑ 20.9% in isokinetic knee flexion peak torque and 18.2% in P_peak_ at 1.05 rad.s(-1) ↑ 10.8% in isokinetic knee extension peak torque at 1.05 rad.s(-1) and 9.4% in isokinetic knee extension peak torque at 4.19 rad.s(-1) ↔ time-of-day dependent variations in strength and power performances even when active^4^ or passive^5^ warm-up conditions were imposed in the morning	Yes	17:30 h	07:30 and 17:30 h
Gauthier *et al*.^77^	1996	Maximal and submaximal voluntary isometric contractions at 90° of elbow flexors	—	n = 7 males n = 6 females	Physical Education students	↑ 3.94% in isometric elbow flexion peak torque at 90° ↑ NME slope throughout the day	Yes	18:00 h	06:00, 09:00, 12:00, 15:00, 18:00, 21:00 and 00:00 h
Giacomoni, Billaut and Falgairette^33^	2006	Maximal voluntary isometric knee extensions	—	n = 12 males	Physically active and healthy adults	↑ 2.1% in 5-min post-test EMG ↑ 14% in 5-min post-test NME (faster short-term recovery patterns of neuromuscular function)	Yes	08:00–10:00 h	08:00–10:00 and 17:00–19:00 h
Gueldich *et al*.^78^	2017	Maximal voluntary isometric knee extensions	5 weeks of training	n = 20 males	Physical Education students	↑ 3.6% and 4.3% in MVC peak torque (in the morning- and evening-training groups, respectively) before the training period ↔ EMG throughout the day ↓ 86% time-of-day dependent variations in MVC peak torque in the morning-training group after 5 weeks of training at a specific time of day	Yes	17:00 h	07:00 and 17:00 h
Guette, Godin and Martin^79^	2005	Plantar flexion of soleus muscle under voluntary and evoked conditions	—	n = 12 males	Physical Education students	↓ 4.9% in MVC peak torque and 18.8% in associated soleus EMG in the evening	Yes	06:00–08:00 h	06:00–08:00 and 17:00–19:00 h
Guette, Gondin and Martin^26^	2005	Electrically evoked and maximal voluntary isometric contractions	—	n = 10 males	Physical Education students	↑ 3.3% in MVC peak torque of the quadriceps and semi-tendinous muscles	Yes	18:18 h^*^	06:00, 10:00, 14:00, 18:00 and 22:00 h
Guette *et al*.^39^	2006	Percutaneous electrical stimuli and maximal voluntary isometric contraction of the plantar flexors	—	n = 11 males	Physical Education students	↓ 7% in MVC peak torque and 21% in associated soleus EMG in the evening	Yes	06:00–08:00 h	06:00–08:00 and 17:00–19:00 h
Kuusmaa, Sedliak and Hakkinen^68^	2015	Maximal bilateral isometric leg press, maximal unilateral isometric knee extension and maximal voluntary activation level during unilateral isometric knee extension	—	n = 72 males	Physically active and healthy adults	↑ 4.4% in MVCLP and 4.3% in MVCKE peak torque at 18:00 h ↑ 10.8% in MVCLP and 5.7% in MVCKE peak torque at 07:30 h in morning-type individuals ↑ 16.1% in MVCLP, 13.5% in MVCKE, 6.2% in MVCVA peak torque and VA% at 18:00 h in evening-type individuals ↔ MVCVA peak torque and VA% throughout the day ↔ EMGLP and EMGVA throughout the day	Yes	-	07:30 and 18:00 h
Küüsmaa-Schildt *et al*.^69^	2017	Maximal voluntary isometric knee extensions coupled with EMG recordings	24 weeks of training	n = 51 males	Physically active and healthy adults	↔ MVC peak torque, P_peak_ and VA% throughout the day after 24 weeks of training at a specific time of day	No	-	06:30–09:30 and 16:30–19:30 h
Lappalainen *et al*.^81^	2009	Isokinetic leg strength (knee extension at 120°/s)	—	n = 26 males	Untrained healthy adults	↑ isokinetic peak torque at 120°/s and total work	Yes	16:30 h	08:00 and 16:30 h
Martin *et al*.^29^	1999	Electrically evoked and maximal voluntary isometric contractions	—	n = 12 males n = 1 female	Healthy adults	↑ 8.9% in MVC peak torque ↑ 9.2% in tetanic force, 18% in maximum rate of tension development and 32% in relaxation of the twitch	Yes	18:00 h	07:00 and 18:00 h
Nicolas *et al*.^82^	2005	Isokinetic leg strength^6^	—	n = 12 males	Physically active and healthy adults	↑ 7.7% in isokinetic peak torque ↓ 3.4–5.1% in NME of vastus lateralis, vastus medialis and rectus femoris muscles in the evening ↔ EMG throughout the day	Yes	18:00 h	06:00 and 18:00 h
Nicolas *et al*.^70^	2007	Isokinetic and isometric leg strength^7^	—	n = 10 males	Physically active and healthy adults	↑ 4–8.8% in MVC peak torque and isokinetic peak torque at 60, 240, 0 and −60°/s	Yes	18:00 h	06:00 and 18:00 h
Nicolas *et al*.^34^	2008	Maximal voluntary isometric knee extensions	—	n = 11 males	Competitive cyclists	↑ 6.7% in MVC peak torque and 6.8% in NME	Yes	18:00 h	06:00 and 18:00 h
Pearson and Onambele^83^	2005	Isokinetic leg strength^8^ coupled with EMG recordings and theta and patella tendon stiffness recordings	—	n = 13 males	N/A	↑ 29.4% in isokinetic knee extension peak torque at 70° ↑ 8% and 35% in vastus lateralis pennation angle in relaxed and peak contracted conditions, respectively ↓ 40% in tendon stiffness in the evening	Yes	17:45 h	07:45 and 17:45 h
Robinson *et al*.^72^	2013	Handgrip strength, isokinetic leg strength^9^ and maximal voluntary isometric contractions	—	n = 10 males	Physically active and healthy adults	↑ 4.3% and 7.6% in left and right handgrip strength, respectively ↑ 10.3% in MVC peak torque ↑ 12.6–16.3% in isokinetic knee flexion and extension peak torque and power at 1.05 rad.s(-1) and 8.6% in isokinetic knee extension peak torque at 4.19 rad.s(-1) ↔ time-of-day dependent variations in strength and power performances when pre-cooling^11^ conditions were imposed in the evening	Yes	17:30 h	07:30 and 17:30 h
Racinais *et al*.^71^	2005	Maximal and submaximal voluntary isometric knee extensions	—	n = 11 males	Physical Education students	↑ 12% in MVC peak torque and 25.4% in muscle contractility ↔ time-of-day dependent variations in strength and power performances in moderately warm and humid^12^ compared with neutral^13^ conditions	Yes	17:00–19:00	07:00–09:00 and 17:00–19:00 h
Sedliak *et al*.^41^	2008	Maximal and submaximal voluntary isometric knee extensions	—	n = 32 males	Physically active and healthy adults^10^	↑ 2.4–8.7% in MVC peak torque at 120°/s throughout the day	Yes	12:00–13:00, 17:00–18:00 and 20:30–21:30 h	07:00–08:00, 12:00–13:00, 17:00–18:00 and 20:30–21:30 h
Sedliak *et al*.^66^	2008	Maximal voluntary isometric knee extensions	10 weeks of training at a specific time of day	n = 34 males	Physically active and healthy adults^10^	↔ magnitude of MVC strength gains after 10 weeks of training at a specific time of day	No	—	09:00–16:00 h
Sedliak *et al*.^73^	2007	Maximal voluntary isometric knee extensions	10 weeks of training at a specific time of day	n = 38 males	Physically active and healthy adults^10^	↑ 3.3–9.2% in MVC peak torque throughout the day before the training period ↔ time-of-day dependent variations in MVC peak torque in the morning-training group after 10 weeks of training at a specific time of day	Yes	12:00, 17:00 and 20:30 h	07:00, 12:00, 17:00 and 20:30 h
Sedliak *et al*.^65^	2009	Maximal voluntary isometric knee extensions	10 weeks of time-of-day-specific resistance training	n = 24 males	Physically active and healthy adults^10^	↔ magnitude of MVC strength gains after 10 weeks of training at a specific time of day	No	—	09:00–16:00 h
Sedliak *et al*.^74^	2018	Maximal voluntary isometric knee extensions	11 weeks of training at a specific time of day	n = 25 males	Physically active and healthy adults^10^	↔ magnitude of MVC strength gains after 11 weeks of training at a specific time of day	No	—	07:30–08:30 and 16:00–17:00 h
Souissi *et al*.^35^	2010	Handgrip strength	—	n = 20 males	Untrained healthy boys	↑ 5.9% in handgrip strength throughout the day	Yes	14:00 and 18:00 h	08:00, 14:00 and 18:00 h
Souissi *et al*.^36^	2012	Maximal voluntary isometric knee extensions	6 weeks of training at a specific time of day	n = 24 males	Untrained healthy boys	↑ 8.4% in MVC peak torque before the training period. ↔ time-of-day dependent variations in MVC peak torque in the morning-training group after 6 weeks of training at a specific time of day	Yes	17:00–18:00 h	07:00–08:00 and 17:00–18:00 h
Souissi *et al*.^54^	2002	Isokinetic leg strength at six angular velocities (1.05, 2.10, 3.14, 4.19, 5.24 and 6.29 rad.s(-1))	6 weeks of training at a specific time of day	n = 14 males	Physical Education students	↑ isokinetic knee extension peak torque at 17:00–18:00 h before the training period. ↔ time-of-day dependent variations in isokinetic knee extension peak torque in the morning-training group after 6 weeks of training at a specific time of day ↑ 38.6% in absolute levels of isometric knee extension peak torque at 07:00–08:00 h in the morning-training group after 6 weeks of training at a specific time of day ↑ 21.2% in absolute levels of isometric knee extension peak torque at 17:00–18:00 h in the evening-training group after 6 weeks of training at a specific time of day	Yes	—	07:00–08:00 and 17:00–18:00 h
Tamm *et al*.^75^	2009	Magnetic stimulation of the cortex, electrical stimulation of the tibial nerve and maximal isometric contractions of the triceps surae muscles	—	n = 16 males n = 7 females	Untrained healthy adults	↑ cortical excitability at 09:00 h in morning-type individuals ↑ cortical excitability at 21:00 h in evening type-individuals ↔ spinal excitability throughout the day in morning- nor evening-type individuals ↔ MVC peak torque nor EMG throughout the day in morning-type individuals. ↑ 13% in MVC peak torque and 23% in EMG throughout the day in evening-type individuals	Yes	—	09:00, 13:00, 17:00 and 21:00 h
Wyse *et al*.^84^	1994	Isokinetic leg strength^14^	—	n = 9 males	Collegiate sportsmen adults	↑ 5–12% in isokinetic peak torque of knee flexors and extensors at 1.05 and 3.14 rad.s(-1)	Yes	18:00–19:30 h	08:00–09:00, 13:00–14:00 and 18:00–19:30 h
Zbidi *et al*.^45^	2016	Maximal voluntary isometric contractions of the elbow flexors and extensors	6 weeks of training at a specific time of day	n = 20 males	Physical Education students	↑ 5.9% and 6.5% in MVF and MRFD, respectively, before the training period ↔ time-of-day dependent variations in MVF and MRFD in the morning-training group after 6 weeks of training at a specific time of day	Yes	17:00–18:00 h	07:00–08:00 and 17:00–18:00 h

EMG electromyographic activity, MCV maximal voluntary contraction, NME Neuromuscular efficiency (mechanical performances/EMG recordings), N/A Not available, ^1^ i.e. knee flexion and extension at 1.05 and 4.19 rad.s(-1) through a 90° range of motion ^2^ i.e. 15–16 h starvation/day; from ≈ 04:00 h till ≈ 19:00 h, ^3^ i.e. 24-h cycling on an indoor trainer with minimal resting periods at a submaximal work rate (paced speed set at 70% of the subject’s maximal aerobic speed, corresponding approximately to 50% of their maximal aerobic power) ^4^i.e. 20–40 min of pedaling on a cycle ergometer at 150 W, ^5^i.e. 45–65 min of resting in a room at 35 °C, 45% relative humidity, ^*^estimated acrophase by cosinor analysis.

EMG electromyographic activity, MCV maximal voluntary contraction, NME Neuromuscular efficiency (mechanical performances/EMG recordings), MVCLP maximal bilateral isometric leg press, EMGLP myoelectric activity during maximal bilateral isometric leg press, MVCKE maximal unilateral isometric knee extension, MVCVA maximal voluntary activation level unilateral isometric knee extension, EMGVA myoelectric activity during maximal voluntary activation level unilateral isometric knee extension, VA% voluntary activation percentage of the quadriceps muscles, ^*^estimated acrophase by cosinor analysis.

EMG electromyographic activity, MCV maximal voluntary contraction, NME Neuromuscular efficiency (mechanical performances/EMG recordings), N/A Not available, ^6^i.e. knee extension at 2.09 rad.s(-1) through a 90° range of motion, ^7^ i.e. knee flexion at 240°/s, 60°/s, 0°/s, −60°/s, ^8^i.e. knee flexion and extension at 90°/s, 80°/s, 70°/s, 50°/s and 30°/s, ^9^ i.e. knee flexion and extension at 1.05 and 4.19 rad.s(-1) through a 90° range of motion, ^10^ i.e. no medication within the last14 days, a non-smoker, regular sleep pattern with sleep duration ranging from 6 to 9 h per night and regular physical activity not more than once a week, ^111^ i.e. immersion in cold water (16.5 °C) before testing to lower rectal temperature to morning values, ^12^i.e. 60 min of resting in a room at 29.5 °C, 74% relative humidity, ^13^ i.e. 60 min of resting in a room at 20.5 °C, 67% relative humidity.

EMG electromyographic activity, MCV maximal voluntary contraction, ^10^i.e. no medication within the last14 days, a non-smoker, regular sleep pattern with sleep duration ranging from 6 to 9 h per night and regular physical activity not more than once a week.

MVF maximal voluntary force, MRFD maximal rate of force development, ^14^i.e. knee flexion and extension at 1.05 and 3.14 rad.s(-1) through a 90° range of motion.

## Discussion

### Effects of time of day on dynamic short-duration maximal exercise performance

In order to explore the influence of time of day on dynamic short-duration maximal exercise performance, several studies have assessed the presence of time-of-day dependent fluctuations in both continuous and intermittent exercises. Dynamic short-duration maximal exercise performance seems to oscillate consistently throughout the day, peaking in the afternoon (i.e. between 16:00 and 20:00 h) with amplitudes ranging from 1.7 to 17.5% (Table 1). Except for two studies^33,46^, better short-duration maximal exercise performances were found in the afternoon when single bouts of exercise were performed under neutral climate conditions. Short-duration maximal exercises that are influenced by the time of day include all-out swimming trials^47–49^, tennis services^37,40^, all-out cycling^13,23,24,27,28,35,36,38,42,43,50–54^, maximal jumps^2,23,36,38,40–42,46,49,55–57^, repeated sprint ability^2,33,44,46,56,58–62^, one repetition maximum (1RM) assessments^63–66^ as well as other force-velocity based tests^35,40,49,54,62,63,67^.

Neuromuscular differences in dynamic short-duration maximal exercise performance throughout the day has been explored using electromyographic (EMG) activity recordings. Castaingts *et al*.^55^ analyzed variations in force and EMG activity of skeletal muscles throughout the day as well as the ratio between these parameters (i.e. force/EMG activity) during a natural movement (i.e. drop jump). Such relationship between the force and EMG activity is called neuromuscular efficiency (NME). In this study, they observed a higher jump height and NME in the evening than in the morning, which indicates that the process of storage-release of potential energy in muscle elastic elements is improved in the evening, without a parallel increase in motor unit activation^55^. A further analysis of time-of-day fluctuations in power and EMG activity of vastus lateralis, rectus femoris, vastus medialis and biceps femoris muscles recorded in a repeated sprint exercise protocol (5 × [6 s of maximal cycling sprint + 30 s of rest]) showed that total work, percentage of peak power decrement and peak power were higher in the evening than in the morning, although it was not accompanied by a time-of-day effect on EMG activity levels^44^. Similarly, Chtourou *et al*.^24^ recorded EMG activity changes during a Wingate anaerobic test and showed that power output and NME were higher in the evening during the first phase of the test (i.e. first 20 s), where peripheral mechanisms of muscle contraction have the main role in exercise performance. Yet, they were independent of the time of day during the second phase of the test (last 10 s), where central mechanisms of muscle contraction have a higher role in exercise performance. Thus, since most studies have shown that changes in muscle function throughout the day are not accompanied by changes in EMG activity levels, it has been suggested that adaptations at the muscle fibre level rather than changes of the neural drive, motor unit properties, and/or muscle membrane properties are more likely to cause time-of-day dependent variations in dynamic short-duration maximal exercise performance^66^.

Other studies have also suggested the existence of peripheral mechanisms which might explain diurnal oscillations in dynamic short-duration maximal exercise performance. For instance, Hammouda *et al*.^58^ observed that the neuromuscular performance during a repeated sprint ability test (5 × [6 s of maximal cycling sprint + 24 s of rest]) was higher in the evening and it was accompanied not only by higher levels of biological markers of muscle injury but also a lower antioxidant status at this time of the day. Furthermore, Racinais *et al*.^61^ assessed time-of-day differences on repeated sprint exercise performance and found a better performance and higher blood lactate concentrations in the evening than in the morning. Although the physiological basis of these time-of-day dependent oscillations has not been elucidated, the evidence suggests the existence of a muscle specific mechanism that accounts for time-of-day dependent fluctuations in neuromuscular performance, whose activity is at least partially independent of the central nervous system.

### Effects of time of day on isometric and isokinetic exercise performance

The performance in isometric and isokinetic exercises fluctuates throughout the day over a wide variety of muscles, with amplitudes ranging from 3 to 29.4% (Table 2). Greater amplitudes in isometric and isokinetic exercise performance were found at around 17:00–19:00 h. These observations included assessments of maximal voluntary isometric contractions^26,29,30,33,34,36,39,41,45,55,65,66,68–80^, isokinetic leg contractions^54,70,72,76,81–84^, handgrip strength^35,72,76^ and electrically evoked contractions^26,29,39,55,75,79^.

The time of day influences isometric and isokinetic exercise performance in both lower and upper extremities. Regarding lower extremities, Guette, Gondin and Martin^26^ found a significant time-of-day effect on maximal voluntary muscle contraction (MVC) peak torque of the quadriceps muscles on the dominant and non-dominant leg, with the highest values occurring at 18:00 h. Regarding upper extremities, Gauthier *et al*.^77^ observed a time-of-day dependent rhythm in elbow flexor torque, whose acrophase was reached at around 18:00 h. This was accompanied by a time-of-day dependent rhythm in biceps muscle EMG activity. Thus, it is clear that upper and lower limb muscle contractility in isometric and isokinetic actions is affected by the time of day. In this sense, most of the studies have shown that when the performance is assessed at a peripheral level by means of peak power, mean power or total work, then a typical peak in performance is found in the evening^29,30,34–36,41,45,54,61,68,70,72,73,76–79,81–85^.

However, the ability to generate force in isometric and isokinetic exercises depends not only on peripheral but also on central mechanisms of muscle contraction. Central mechanisms include central nervous system command, alertness and motivation, being them all normally assessed by electromyographic activity recording of skeletal muscles^68^. When muscle performance is assessed at central level, higher morning values^77,79^, higher evening values^80^ or no differences throughout the day^26,29,66,82^ are observed. Such differences in EMG activity throughout the day observed among different studies are difficult to be explained. However, many researchers suggest that EMG activity recordings can be affected by the muscle groups examined. Strikingly, time-of-day dependent fluctuations in maximal isometric and isokinetic performance seem to be different between fast and slow muscles of the lower limbs. In this sense, a decrease in MVC peak torque of triceps surae muscles and soleus muscle EMG activity was observed in the evening in comparison to the morning. According to the authors, this is probably due to a higher fatigue state of the slow motor units as the day progresses^39,79^. It is worth noting that methodological factors associated with EMG activity recordings might also account for differences in central mechanisms of muscle contraction throughout the day.

Finally, some studies have suggested that diurnal fluctuations in short-duration maximal exercise performance may be partially controlled by the individual circadian typology/chronotype. Thus, inter-individual differences related to the chronotype might generate opposite responses in central and peripheral mechanisms of muscle contraction throughout the day. Chronotype is an individual’s characteristic pattern that reflects preferences towards morningness or eveningness, and it is usually evaluated using self-assessment questionnaires. The effect of the chronotype on isometric and dynamic exercise performance has not been extensively studied, but some studies have shown the importance of the individual typology on isometric exercise performance. For instance, Kuusmaa *et al*.^68^ showed that morning-type (M-type) individuals exhibited lower force values in the evening whereas evening-type (E-type) individuals exhibited lower force values in the morning, despite the fact that EMG activities where not different throughout the day in either group. Tamm *et al*.^75^ reported that E-type individuals showed parallel increases in cortical and spinal excitability over the day, which were reflected on an increased triceps surae EMG activity and MVC torque at 21:00 h. However, M-type individuals exhibited the highest cortical excitability at 09:00 h but the highest spinal excitability at 21:00 h, without significant differences in triceps surae EMG activity or torque produced during MVCs over the day. Such discrepancies have led some scientists to the conclusion that EMG activity and MVC torque could increase, remain constant or even decrease throughout the day, thus reflecting an orchestrated response between peripheral and central mechanisms in the control of skeletal muscle contractile properties^55^.

### General mechanisms of time-of-day effects on short-duration maximal exercise performance

Performances in short-duration maximal exercises follow a time-of-day dependent rhythmicity, peaking in the afternoon at around 16:00–20:00 h. This effect was observed in children and adults^35,66^ and among different sports disciplines^40,49,50^. Diurnal amplitudes in short-duration maximal exercise performance range from 1.7 to 29.4% depending on the muscle feature, muscle group and assessment method. According to the scientific literature published to date, it is clear that a better short-duration maximal exercise performance is achieved in the evening than in the morning in both dynamic^13,24,27,28,30,35–38,40–44,47–61,67,85–90^ and isometric/isokinetic^26,29,30,33–36,39,41,45,54,55,68,70–73,75–85^ exercise modes. However, these differences may be blunted in warm and humid environments^56,62^. This also happens when appropriate warm-up protocols are used^57,59^ or if music is listened to during warm-ups^13,86^. The same effect may take place if intermittent fasting conditions are imposed^50,85^ or if regular exercise training is performed at a specific time of day^30,36,45,54,63–66,69,73,74,78^.

#### Effects of temperature

Since short-duration maximal exercise performance fluctuates in parallel with body temperature^56,62^, it has therefore been suggested that body temperature affects the contractile properties, viscosity and conduction velocity of action potentials in skeletal muscle as well as the extensibility of connective tissue^91^. In fact, Racinais and Oksa^92^ have found a positive and linear relationship between performance and muscle temperature, where performance improves from 2 to 5% with a 1 °C increase in muscle temperature. Hence, several research groups have been devoted to studying the effects of getting exposed to warm or cold environments on short-duration maximal exercise performance at different times of the day. In this sense, it was observed that a 60-min exposure to a moderately warm and humid environment (i.e. 28.1–29.5 °C, 62.6–74% relative humidity) blunts the diurnal variation in muscle function that is observed in neutral climate conditions (i.e. 20–20.5 °C, 57–67% relative humidity)^56,62,71^. Conversely, immersion in cold water (i.e. 16–16.5 °C) before performance testing in the evening has shown to reduce the force and muscle power towards morning values^60,72^. This is achieved through a “passive effect” of thermal conditions on muscle temperature^92^. In addition to the passive effect of climate conditions on body temperature, time-of-day dependent variations in muscle performance might also diminish or even disappear when extended warm-up protocols are used (i.e. 20 min cycling on a stationary bike at 150–200 W prior to the completion of the regular warm-up)^57^. Diurnal fluctuations in performance also disappear under active (i.e. 12 min of pedaling at 50% of VO_2max_ interspersed with three brief accelerations of 5 s) but not passive (i.e. 3 min of pedaling at 70 rpm at 50% of VO_2max_) warm-up conditions^59^. The same can be stated when longer active warm-ups (i.e. 15 min of pedaling at 50% of maximal power output) are compared with shorter ones (i.e. 5 min of pedaling at 50% of maximal power output)^42^. The effectiveness of these protocols to increase performance in the morning towards evening values is due to an “active effect” on muscle temperature^92^. Thus, increases in muscle temperature might help athletes to minimize diurnal dependent variations in short-duration maximal exercise performance. This can be achieved by means of short exposures to warm and humid environments as well as by the inclusion of active warm-up protocols (e.g. 12–15 min pedaling at minimum intensities of 50% VO2max and interspersed with sprint exercises) before exercising.

Finally, although there is strong evidence to support that time-of-day dependent fluctuations in short-duration maximal exercise are dependent on body temperature, it is clear that there are additional factors which might affect such fluctuations. In this sense, it should be noted that in moderately warm and humid climate conditions better performances are achieved in the morning, when body temperature is at its lowest. However, this is not observed in the evening^56,62,71^. In this regard, it seems that the effects of the time of day on body temperature and those of warm and humid climate conditions do not interact to potentiate exercise performance in the evening. According to some authors, this is due to the similar effects of both variables on neuromuscular efficiency^59^, although there is no data to support this hypothesis. On the other hand, it has been observed that an increase in rectal morning temperature towards evening values does not increase muscle strength levels in the morning towards levels found in the evening^76,87,89^. This might suggest that a specific mechanism within the muscle is orchestrating the effects of time of day on short-duration maximal exercise performance. It is also worth mentioning that when passive exposure to hot environments increases the central temperature towards values close to 39 °C, there is a reduction in the neural drive and the maximal voluntary activation of muscles. This will negatively affect power production and short-duration maximal exercise performance^92^.

#### Effects of music

In addition to peripheral factors, it has been suggested that central factors related to alertness, motivation and mood might affect short-duration maximal exercise performance at different times of the day^13,28,33,86,93,94^. It has been well established that an auditory stimulus, such as music, is effective in increasing levels of arousal, reducing levels of perceived exertion and facilitating a better motor coordination during short-duration maximal exercises^94–98^. In this regard, Chtourou *et al*.^13^ observed higher performances in the Wingate anaerobic test when it was performed after *warm-up with music* [10-min warm-up while listening high tempo music (>120–140 bpm) through headphones] in comparison to *warm-up without music* conditions. Furthermore, listening to music during the warm-up period blunted time-of-day dependent variations in muscle power output. This effect was achieved due to a greater increase in performance in the morning than in the evening. Interestingly, a recent study carried out by the same research group showed that this beneficial effect of music on short-duration maximal exercise performance can be achieved when the 10-min warm-up is performed either under *neutral* (i.e. non-selected) or *self-selected motivational* music conditions^86^. In the same study, a greater effect of music on short-duration maximal exercise performance in the morning than in the evening was reported. According to the authors, the ergogenic effect of music could be related to better individual perceptions of self-esteem and sense of confidence in the morning, where the level of motivation seems to be lower among individuals. Thus, listening to music during the warm-up period could be an effective strategy to improve short-duration maximal exercise performance, especially in the morning hours.

#### Effects of testosterone and cortisol

Short-duration maximal exercises produce an acute increase in both serum testosterone and cortisol levels, affecting the anabolic and catabolic status in skeletal muscle^99^. In addition, testosterone and cortisol are under circadian regulation, both peaking in the morning hours^100^. This has raised the question of whether the exercise-induced hormonal response can be influenced by the time of day. In this regard, Bird and Tarpenning^101^ found that a single session of heavy resistance exercise produced a lower cortisol response when performed in the evening compared with the morning, without affecting the diurnal rhythmicity of testosterone. This suppression in the cortisol response after resistance training in the evening hours was also observed by Burley *et al*.^102^. The authors proposed that this reduction in the testosterone/cortisol ratio leads to a reduced catabolic environment which could favor muscle adaptations to resistance exercise in the evening hours. Interestingly, Sedliak *et al*.^73^ observed that a 10-week training period at a specific time of day gradually reduced morning but not evening cortisol levels. The authors attributed these changes to a lower anticipatory stress and a better accommodation to early waking rather than any chronic change in cortisol diurnal rhythmicity. However, this period of training at a specific time of day was enough to blunt the time-of-day effects on strength performance in these individuals. Moreover, the absolute increase in maximum strength at the end of the training period was similar in all individuals regardless the time of day at which training was conducted.

It is worth mentioning that, although some studies have observed a relationship between hormone levels, short-duration maximal exercise performance and the time of day, there are other ones that have not so^63,64,74^. For instance, Kūūsma *et al*.^64^ observed no changes in the typical diurnal variations in resting serum testosterone and cortisol concentrations after a 24-week period of training at a specific time of day. According to the authors, the effects of a temporary phase shift caused after a prolonged period of training might explain these results. Furthermore, Sedliak *et al*.^74^ observed that resting cortisol levels declined independently of the time of day after 11 weeks of training at a specific time of day in untrained young males. However, they did not find changes in resting testosterone levels after the training period in such individuals. Likewise, no significant changes were observed in resting testosterone levels in a group of healthy older women after 12 weeks of training at a specific time of day^63^. In summary, whereas resistance exercise training periods at a specific time of day do not seem to affect the circadian pattern of testosterone secretion, their effect on cortisol secretion diurnal pattern is more controversial. Therefore, further research is required to elucidate the role of the time of day on the exercise-induced hormonal response.

#### Effects of circadian systems

Recently, a large-scale transcriptomic analysis has revealed the existence of rhythmic and CLOCK-driven pathways in human skeletal muscle that affect ~8% of muscle genes^103^. This analysis showed that the transcription of human skeletal muscle clock genes was distributed into two phases of transcript accumulation at 04:00 and 16:00 h. The afternoon peak was enriched in genes related to muscle contraction and mitochondrial activity whereas the early morning peak was enriched in genes associated with inflammation and the immune response^103^. In addition, the use of genomic techniques has led to the identification of metabolic pathways regulated by clock genes within skeletal muscle, including those involved in glucose uptake, lipid metabolism and myokine secretion^104–106^. Dyar *et al*.^104^ observed that skeletal muscles of mice with a specific ablation of the core clock gene *Bmal1* present an impaired insulin-stimulated glucose uptake due to (1) reduced protein levels of the insulin-dependent glucose transporter GLUT4 and the Rab-GTPase TBC1D1 involved in GLUT4 translocation to the plasma membrane and (2) a decrease in the activity of the glycolytic enzyme PDH. Furthermore, a high-resolution microarray analysis in a muscle-specific *Bmal1* knockout mice model revealed a temporal separation of genes involved in carbohydrates and fatty acids use and storage over a period or circadian time of 24 h (CT 1 to 24)^107^. In this sense, circadian expression of genes involved in carbohydrate catabolism such as hexokinase-2 (*Hk2*; which catalyzes the first step of glycolysis) and pyruvate dehydrogenase phosphatase (*Pdp1*; which activates PDH) peak in the late inactive and early active phases of the day (CT 10–12). Moreover, the pyruvate dehydrogenase kinase gene (*Pdk4*; which inhibits PDH) peaks in the middle of the inactive phase of the day (CT 6). This circadian pattern of gene expression might promote an increase in the glycolytic flux during the active phase of the day (CT 12–24)^107^. On the other hand, genes involved in fatty acid uptake and β-oxidation such as acyl-carnitine translocase (*Slc25a20*; which transfers fatty acids into the mitochondrial matrix) and malonyl-CoA decarboxylase (*Mlycd*; which promotes β-oxidation by reducing malonyl-CoA levels) reach their peak expression in the middle of the inactive phase of the day (CT 7.5). Moreover, the gene that encodes for the nuclear PPARG co-activator 1 beta (*Ppargc1b*) which stimulates the activity of several transcription factors involved in mitochondrial biogenesis, fatty acid uptake and β-oxidation, also peaks in the middle of the inactive phase of the day (CT 7)^107^. Altogether, these results could suggest that there is a shift in the use of substrates from lipids to carbohydrates. This metabolic shift, which depends on the circadian gene expression in skeletal muscle, goes from the inactive (CT 1–12) to the active (CT 12–24) phase of the day. This circadian pattern of metabolic gene expression in skeletal muscle could explain the greater performance in short-duration maximal exercises observed in the evening hours (i.e. between 16:00 and 20:00 h). In this sense, a greater activation of the glycolytic pathway in the active phase of the day would promote greater performances in anaerobic exercises in the evening hours.

In spite of the typical temporary peak observed in the evening hours, many studies have demonstrated that regular training at a specific time of the day is able to blunt the diurnal fluctuations in maximal exercise performance^30,36,45,54,63–66,69,73,74,78^. The mechanisms responsible for the adaptations of training at a specific time of day in short-duration maximal exercise performance remain unknown. However, Sedliak *et al*.^66^ observed that the EMG activity during a maximal voluntary contraction of the knee extensors did not show any time-of-day specific adaptation after a 10-week training period at a specific time of day. This led the authors to suggest that peripheral rather than neural adaptations are the main source of temporal specificity in strength training. This could be caused by (1) an attenuation in training adaptations throughout the 10-week training period or (2) individual chronotype-related differences in responsiveness to training at a specific time of day. Likewise, intermittent fasting conditions, such as those imposed during the Ramadan period (i.e. 15–16 h starvation/day; from ≈ 04:00 h until ≈ 19:00 h) have shown to exert the same effect, minimizing the time-of-day dependent variations in exercise performance^50,85^. Chtourou *et al*.^50^ have suggested that Ramadan might act directly on the circadian rhythm of anaerobic performance by means of (1) inducing a phase advance or delay in the rhythm or (2) reducing the amplitude of the rhythm of the anaerobic power development. Despite the fact that none of these hypotheses have been tested to date, some of them have raised the question as whether circadian systems could account for time-of-day specific training adaptations.

There is evidence that scheduled exercise is able to cause phase shifts in the circadian system within skeletal muscle in mice^15,16^. Using a PER2::LUC circadian reporter mouse model, Wolff and Esser^15^ showed that four weeks of either voluntary or involuntary exercise for 2 hours/day were able to shift the phase of locomotor and molecular rhythms in skeletal muscle towards an earlier time of day. Furthermore, Edgar and Dement^16^ used two different wheel restriction schedules to determine whether exercise duration was an important determinant of feedback activity to the mouse circadian system. In this study, they found that more than 75% of total wheel activity occurred in the initial 2 hours over a 6-h or 12-h period of wheel availability. This led the authors to suggest that exercise intensity might be a primary determinant of the exercise-dependent phase shift strength. Thus, the ability of exercise to provide temporal feedback to the circadian system could explain why time-of-day effects on short-maximal exercise performance are blunted after a training period at a specific time of day. In humans, Zambon *et al*.^17^ found that the expression of circadian clock genes was affected in quadriceps muscles at 6 and 18 h after having performed a single session of one-leg resistance exercise. In this study, resistance exercise shifted the expression patterns of diurnal-regulated genes through two mechanisms: (1) by upregulating genes that are normally repressed in the morning; and (2) by downregulating genes that are normally activated in the morning. Among them, it was observed an upregulation of the gene that encodes for a regulatory subunit of protein phosphatase-1 (*Ppp1r5*), which is normally repressed in the morning. Protein phosphatase-1 is responsible for activating glycogen synthase while also inhibiting glycogen breakdown. Moreover, it was observed a downregulation of the gene that encodes for the mitochondrial uncoupling protein 3 (*Ucp3*), which is upregulated in the morning. Uncoupling protein 3 stimulates oxidative phosphorylation by creating proton leaks across the inner mitochondrial membrane. These data show that exercise represents an important circadian time cue and changes the phase of the molecular clock, specifically in peripheral tissues. Altogether, these results suggest that skeletal muscle molecular clocks might account for the adaptations of training at a specific time of day, suggesting a link between molecular clocks in skeletal muscle and exercise performance throughout the day.

### Practical applications

Based on the studies summarized in this manuscript, it is clear that the time of day at which short-duration maximal exercise is conducted is an important variable for training prescription. However, to date, there are no guidelines to help athletes or coaches to achieve optimal performances or avoid deleterious effects of time of day on short-duration maximal exercise performance. Accordingly, some general remarks in this respect include: (1) under neutral climate conditions (i.e. 20–20.5 °C, 57–67% relative humidity), better short-duration maximal exercise performances are achieved in the afternoon (i.e. between 16:00 and 20:00 h) compared with the morning; (2) time-of-day deleterious effects on short-duration maximal exercise performance in the morning may be minimized after: (a) a 10-min warm-up period while listening to neutral or self-selected high-tempo music (>120–140 bpm) through headphones; (b) a 60-min exposure to warm and humid climate conditions (i.e. 28.1–29.5 °C, 62.6–74% relative humidity); (c) active warm-up protocols (e.g. 12–15 min pedaling at minimum intensities of 50% VO2max and interspersed with 5-s sprint exercises); (d) 2–4 weeks of intermittent fasting conditions of 15–16 h starvation/day from ≈04:00 h till ≈19:00 h (e) a training period of at least 5 weeks performed in the morning.

## Conclusion

In summary, this review shows that, under neutral climate conditions, short duration maximal exercise performance is affected by the time of day, peaking between 16:00 and 20:00 h. However, a similar performance may be achieved in the morning hours if exercise is conducted after: (1) short exposures to moderately warm and humid environments; (2) active warm-up protocols; (3) intermittent fasting conditions; (4) warming-up while listening to music; (5) prolonged periods of training at a specific time of day. This suggests that time-of-day dependent fluctuations in short-duration maximal exercise performance are controlled not only by body temperature, hormone levels, motivation or mood states but also by a versatile circadian system within skeletal muscle.

## Supplementary information

Supplementary Information.

## Data Availability

The datasets generated during and/or analyzed during the current study are available in the MEDLINE and Google Scholar repositories, https://www.ncbi.nlm.nih.gov/pubmed/, https://scholar.google.com/, respectively.

## References

[1] Liu AC, Lewis WG, Kay SA (2007). Mammalian circadian signaling networks and therapeutic targets. Nature Chemical Biology.

[2] Racinais S (2006). Effect of an acute hot and dry exposure in moderately warm and humid environment on muscle performance at different times of day. Int J Sports Med.

[3] Mohawk JA, Takahashi JS (2011). Cell autonomy and synchrony of suprachiasmatic nucleus circadian oscillators. Trends Neurosci.

[4] Partch CL, Green CB, Takahashi JS (2014). Molecular architecture of the mammalian circadian clock. Trends Cell Biol.

[5] Kimberly HC, Joseph ST (2019). Circadian clock genes and the transcriptional architecture of the clock mechanism. Journal of Molecular Endocrinology.

[6] Kennaway DJ (2007). Metabolic homeostasis in mice with disrupted Clock gene expression in peripheral tissues. Am J Physiol Regul Integr Comp Physiol.

[7] Kohsaka A (2007). High-fat diet disrupts behavioral and molecular circadian rhythms in mice. Cell Metab.

[8] Vieira E, Burris TP, Quesada I (2014). Clock genes, pancreatic function, and diabetes. Trends Mol Med.

[9] Vieira E (2012). The clock gene Rev-erbalpha regulates pancreatic beta-cell function: modulation by leptin and high-fat diet. Endocrinology.

[10] Vieira E (2013). Involvement of the clock gene Rev-erb alpha in the regulation of glucagon secretion in pancreatic alpha-cells. PLoS One.

[11] Balsalobre A, Damiola F, Schibler U (1998). A serum shock induces circadian gene expression in mammalian tissue culture cells. Cell.

[12] Ko CH, Takahashi JS (2006). Molecular components of the mammalian circadian clock. Hum Mol Genet.

[13] Chtourou H (2012). Listening to music affects diurnal variation in muscle power output. Int J Sports Med.

[14] Monk TH (2010). Enhancing circadian zeitgebers. Sleep.

[15] Wolff G, Esser KA (2012). Scheduled exercise phase shifts the circadian clock in skeletal muscle. Med Sci Sports Exerc.

[16] Edgar DM, Dement WC (1991). Regularly scheduled voluntary exercise synchronizes the mouse circadian clock. American Journal of Physiology-Regulatory, Integrative and Comparative Physiology.

[17] Zambon AC (2003). Time- and exercise-dependent gene regulation in human skeletal muscle. Genome biology.

[18] Manfredini R (1998). Circadian rhythms, athletic performance, and jet lag. Br J Sports Med.

[19] Winget CM, DeRoshia CW, Holley DC (1985). Circadian rhythms and athletic performance. Med Sci Sports Exerc.

[20] Tahara Y, Shibata S (2018). Entrainment of the mouse circadian clock: Effects of stress, exercise, and nutrition. Free Radic Biol Med.

[21] Tahara Y, Aoyama S, Shibata S (2017). The mammalian circadian clock and its entrainment by stress and exercise. J Physiol Sci.

[22] Sato, S. *et al*. Time of Exercise Specifies the Impact on Muscle Metabolic Pathways and Systemic Energy Homeostasis. *Cell Metab* (2019). 10.1016/j.cmet.2019.03.013 31006592

[23] Ezagouri, S. *et al*. Physiological and Molecular Dissection of Daily Variance in Exercise Capacity. *Cell Metab*, (2019). 10.1016/j.cmet.2019.03.012 31006590

[24] Chtourou H (2011). Diurnal variation in Wingate-test performance and associated electromyographic parameters. Chronobiol Int.

[25] Facer-Childs E, Brandstaetter R (2015). The impact of circadian phenotype and time since awakening on diurnal performance in athletes. Curr Biol.

[26] Guette M, Gondin J, Martin A (2005). Time-of-day effect on the torque and neuromuscular properties of dominant and non-dominant quadriceps femoris. Chronobiol Int.

[27] Hammouda O (2012). High intensity exercise affects diurnal variation of some biological markers in trained subjects. Int J Sports Med.

[28] Lericollais R (2009). Time-of-day effects on fatigue during a sustained anaerobic test in well-trained cyclists. Chronobiol Int.

[29] Martin A (1999). Effect of time of day on force variation in a human muscle. Muscle Nerve.

[30] Chtourou H (2012). The effect of training at the same time of day and tapering period on the diurnal variation of short exercise performances. J Strength Cond Res.

[31] Buchheit, M. & Laursen, P. High-Intensity Interval Training, Solutions to the Programming Puzzle: Part II: Anaerobic Energy, Neuromuscular Load and Practical Applications. Sports medicine (Auckland, N.Z.), **43** (2013). 10.1007/s40279-013-0066-5 23832851

[32] Gastin PB (2001). Energy system interaction and relative contribution during maximal exercise. Sports Med.

[33] Giacomoni M, Billaut F, Falgairette G (2006). Effects of the time of day on repeated all-out cycle performance and short-term recovery patterns. Int J Sports Med.

[34] Nicolas, A. *et al*. Effect of time-of-day on neuromuscular properties of knee extensors after a short exhaustive cycling exercise. *Isokinetics and exercise science*, **16** (2008).

[35] Souissi H (2010). Time-of-Day Effects on Short-Term Exercise Performances in 10- to 11-Year-Old Boys. Pediatric Exercise Science.

[36] Souissi H (2012). The effect of training at a specific time-of-day on the diurnal variations of short-term exercise performances in 10- to 11-year-old boys. Pediatr Exerc Sci.

[37] Atkinson G, Speirs L (1998). Diurnal Variation in Tennis Service. Perceptual and Motor Skills.

[38] Bernard T (1998). Time-of-day effects in maximal anaerobic leg exercise. Eur J Appl Physiol Occup Physiol.

[39] Guette M (2006). Plantar flexion torque as a function of time of day. Int J Sports Med.

[40] López-Samanes Á (2017). Circadian rhythm effect on physical tennis performance in trained male players. Journal of Sports Sciences.

[41] Sedliak M (2008). Diurnal variation in maximal and submaximal strength, power and neural activation of leg extensors in men: multiple sampling across two consecutive days. Int J Sports Med.

[42] Souissi N (2010). Diurnal variation in Wingate test performances: influence of active warm-up. Chronobiol Int.

[43] Souissi N (2004). Circadian rhythms in two types of anaerobic cycle leg exercise: force-velocity and 30-s Wingate tests. Int J Sports Med.

[44] Zarrouk N (2012). Time of day effects on repeated sprint ability. Int J Sports Med.

[45] Zbidi S (2016). Diurnal Rhythm of Muscular Strength Depends on Temporal Specificity of Self-Resistance Training. J Strength Cond Res.

[46] Blonc S (2010). Effects of 5 weeks of training at the same time of day on the diurnal variations of maximal muscle power performance. J Strength Cond Res.

[47] Arnett MG (2002). Effects of prolonged and reduced warm-ups on diurnal variation in body temperature and swim performance. Journal of strength and conditioning research.

[48] Baxter C, Reilly T (1983). Influence of time of day on all-out swimming. British journal of sports medicine.

[49] Pallarés, J. *et al*. Circadian rhythm effects on neuromuscular and sprint swimming performance. Vol. 45. 2014.

[50] Chtourou H (2012). The effect of time-of-day and Ramadan fasting on anaerobic performances. Int J Sports Med.

[51] Hill DW (1992). Effect of time of day on aerobic and anaerobic responses to high-intensity exercise. Can J Sport Sci.

[52] Lericollais R (2011). Diurnal evolution of cycling biomechanical parameters during a 60-s Wingate test. Scand J Med Sci Sports.

[53] Melhim AF (1993). Investigation of circadian rhythms in peak power and mean power of female physical education students. Int J Sports Med.

[54] Souissi N (2002). Effects of regular training at the same time of day on diurnal fluctuations in muscular performance. J Sports Sci.

[55] Castaingts V (2004). Neuromuscular efficiency of the triceps surae in induced and voluntary contractions: morning and evening evaluations. Chronobiol Int.

[56] Racinais S, Hue O, Blonc S (2004). Time-of-day effects on anaerobic muscular power in a moderately warm environment. Chronobiol Int.

[57] Taylor K (2011). Warm-up affects diurnal variation in power output. Int J Sports Med.

[58] Hammouda O (2011). Diurnal variations of plasma homocysteine, total antioxidant status, and biological markers of muscle injury during repeated sprint: effect on performance and muscle fatigue–a pilot study. Chronobiol Int.

[59] Racinais S, Blonc S, Hue O (2005). Effects of active warm-up and diurnal increase in temperature on muscular power. Med Sci Sports Exerc.

[60] Racinais S (2009). Does the diurnal increase in central temperature interact with pre-cooling or passive warm-up of the leg?. J Sci Med Sport.

[61] Racinais S (2005). Morning versus evening power output and repeated-sprint ability. Chronobiol Int.

[62] Racinais S (2004). Time-of-day effects in maximal anaerobic leg exercise in tropical environment: a first approach. Int J Sports Med.

[63] Krčmárová B (2018). The effects of 12-week progressive strength training on strength, functional capacity, metabolic biomarkers, and serum hormone concentrations in healthy older women: morning versus evening training. Chronobiology International.

[64] Kuusmaa M (2016). Effects of morning versus evening combined strength and endurance training on physical performance, muscle hypertrophy, and serum hormone concentrations. Appl Physiol Nutr Metab.

[65] Sedliak M (2009). Effect of time-of-day-specific strength training on muscular hypertrophy in men. J Strength Cond Res.

[66] Sedliak M (2008). Effect of time-of-day-specific strength training on maximum strength and EMG activity of the leg extensors in men. J Sports Sci.

[67] Javierre C (1996). Influence of sleep and meal schedules on performance peaks in competitive sprinters. Int J Sports Med.

[68] Kuusmaa M, Sedliak M, Hakkinen K (2015). Effects of time-of-day on neuromuscular function in untrained men: Specific responses of high morning performers and high evening performers. Chronobiol Int.

[69] Kuusmaa-Schildt M (2017). Neuromuscular Adaptations to Combined Strength and Endurance Training: Order and Time-of-Day. Int J Sports Med.

[70] Nicolas A (2007). Effect of circadian rhythm of neuromuscular properties on muscle fatigue during concentric and eccentric isokinetic actions. Isokinetics and Exercise Science.

[71] Racinais S (2005). Time of day influences the environmental effects on muscle force and contractility. Med Sci Sports Exerc.

[72] Robinson WR (2013). Does lowering evening rectal temperature to morning levels offset the diurnal variation in muscle force production?. Chronobiol Int.

[73] Sedliak M (2007). Effect of time-of-day-specific strength training on serum hormone concentrations and isometric strength in men. Chronobiol Int.

[74] Sedliak M (2018). Morphological, molecular and hormonal adaptations to early morning versus afternoon resistance training. Chronobiol Int.

[75] Tamm AS (2009). Chronotype influences diurnal variations in the excitability of the human motor cortex and the ability to generate torque during a maximum voluntary contraction. J Biol Rhythms.

[76] Edwards BJ (2013). Does raising morning rectal temperature to evening levels offset the diurnal variation in muscle force production?. Chronobiol Int.

[77] Gauthier A (1996). Diurnal rhythm of the muscular performance of elbow flexors during isometric contractions. Chronobiol Int.

[78] Gueldich H (2017). Electrostimulation Training Effects on diurnal Fluctuations of Neuromuscular Performance. Int J Sports Med.

[79] Guette M, Gondin J, Martin A (2005). Morning to evening changes in the electrical and mechanical properties of human soleus motor units activated by H reflex and M wave. Eur J Appl Physiol.

[80] Callard D (2000). Circadian rhythms in human muscular efficiency: continuous physical exercise versus continuous rest. A crossover study. Chronobiol Int.

[81] Lappalainen Z (2009). Time-of-day effects during acute isokinetic exhaustive eccentric exercise: Serum leptin response. Isokinetics and Exercise Science.

[82] Nicolas A (2005). Time-of-day effects on myoelectric and mechanical properties of muscle during maximal and prolonged isokinetic exercise. Chronobiol Int.

[83] Pearson SJ, Onambele GN (2005). Acute changes in knee-extensors torque, fiber pennation, and tendon characteristics. Chronobiol Int.

[84] Wyse JP, Mercer TH, Gleeson NP (1994). Time-of-day dependence of isokinetic leg strength and associated interday variability. Br J Sports Med.

[85] Aloui A (2013). Effects of Ramadan on the diurnal variations of repeated-sprint performances. Int J Sports Physiol Perform.

[86] Belkhir Y (2019). Listening to neutral or self-selected motivational music during warm-up to improve short-term maximal performance in soccer players: Effect of time of day. Physiol Behav.

[87] Pullinger S (2019). Effects of an active warm-up on variation in bench press and back squat (upper and lower body measures). Chronobiol Int.

[88] Robertson CM (2018). Is the diurnal variation in muscle force output detected/detectable when multi-joint movements are analysed using the musclelab force-velocity encoder?. Chronobiol Int.

[89] Pullinger SA (2018). Diurnal variation in repeated sprint performance cannot be offset when rectal and muscle temperatures are at optimal levels (38.5 degrees C). Chronobiol Int.

[90] West DJ (2014). The influence of the time of day on core temperature and lower body power output in elite rugby union sevens players. J Strength Cond Res.

[91] Shephard RJ (1984). Sleep, Biorhythms and Human Performance. Sports Medicine.

[92] Racinais S, Oksa J (2010). Temperature and neuromuscular function. Scandinavian Journal of Medicine & Science in Sports.

[93] Reilly T, Down A (1992). Investigation of circadian rhythms in anaerobic power and capacity of the legs. J Sports Med Phys Fitness.

[94] Chtourou H, Hmida C, Souissi N (2017). Effect of music on short-term maximal performance: sprinters vs. long distance runners. Sport Sciences for Health.

[95] Simpson SD, Karageorghis CI (2006). The effects of synchronous music on 400-m sprint performance. J Sports Sci.

[96] Yamashita S (2006). Effects of music during exercise on RPE, heart rate and the autonomic nervous system. J Sports Med Phys Fitness.

[97] Eliakim M (2007). The effect of music during warm-up on consecutive anaerobic performance in elite adolescent volleyball players. Int J Sports Med.

[98] Pujol TJ, Langenfeld ME (1999). Influence of music on Wingate Anaerobic Test performance. Percept Mot Skills.

[99] Kraemer WJ, Ratamess NA (2005). Hormonal responses and adaptations to resistance exercise and training. Sports Med.

[100] Piro C (1973). Circadian rhythm of plasma testosterone, cortisol and gonadotropins in normal male subjects. Journal of Steroid Biochemistry.

[101] Bird SP, Tarpenning KM (2004). Influence of circadian time structure on acute hormonal responses to a single bout of heavy-resistance exercise in weight-trained men. Chronobiol Int.

[102] Burley SD (2016). The Differential Hormonal Milieu of Morning versus Evening May Have an Impact on Muscle Hypertrophic Potential. PLoS One.

[103] Perrin L (2018). Transcriptomic analyses reveal rhythmic and CLOCK-driven pathways in human skeletal muscle. eLife.

[104] Dyar KA (2014). Muscle insulin sensitivity and glucose metabolism are controlled by the intrinsic muscle clock. Molecular Metabolism.

[105] Perrin L (2015). Human skeletal myotubes display a cell-autonomous circadian clock implicated in basal myokine secretion. Molecular Metabolism.

[106] van Moorsel D (2016). Demonstration of a day-night rhythm in human skeletal muscle oxidative capacity. Molecular metabolism.

[107] Hodge BA (2015). The endogenous molecular clock orchestrates the temporal separation of substrate metabolism in skeletal muscle. Skeletal Muscle.

