# Supplementary material for: Time-of-Day Effects on Short-Duration Maximal Exercise Performance
Source: Sci Rep. 2020 Jun 11;10:9485. doi: 10.1038/s41598-020-66342-w (PMC7289891; doi:10.1038/s41598-020-66342-w)
Supplement: Supplementary file 1 — Supplementary Information. [file 41598_2020_66342_MOESM1_ESM.pdf]

# Supplementary material

**Search strategy**

PubMed and PubMed Central (184 results)

(“time of day” OR “time-of-day”) AND (exercise performance) AND (morning OR evening OR afternoon OR night) AND (strength OR neuromuscular OR resistance OR endurance OR isometric OR isokinetic))

Google Scholar (352 results)

("time-of-day" OR "time of day") AND ("exercise performance") AND ("short-term exercise" OR "short-duration exercise") AND (morning OR evening OR afternoon OR night) AND (strength OR neuromuscular OR resistance OR endurance OR isometric OR isokinetic)
